# MYCN is a novel oncogenic target in pediatric T-cell Acute Lymphoblastic Leukemia

**DOI:** 10.18632/oncotarget.1337

**Published:** 2013-09-29

**Authors:** Annalisa Astolfi, Francesca Vendemini, Milena Urbini, Fraia Melchionda, Riccardo Masetti, Monica Franzoni, Virginia Libri, Salvatore Serravalle, Marco Togni, Giuseppina Paone, Luca Montemurro, Daniela Bressanin, Francesca Chiarini, Alberto M. Martelli, Roberto Tonelli, Andrea Pession

**Affiliations:** ^1^ “Giorgio Prodi” Cancer Research Center, University of Bologna, Bologna, Italy;; ^2^ Pediatric Oncology and Hematology Unit “Lalla Seràgnoli”, S. Orsola-Malpighi Hospital, University of Bologna, Bologna, Italy;; ^3^ Department of Biomedical and Neuromotor Sciences, University of Bologna, Bologna, Italy;; ^4^ Institute of Molecular Genetics, National Research Council-IOR, Bologna, Italy;; ^5^ Department of Pharmacy and Biotechnology, University of Bologna, Bologna, Italy.

**Keywords:** pediatric T-ALL, MYCN, peptide nucleic acid, TAL1, TGF-β inhibitors

## Abstract

MYCN is an oncogene frequently overexpressed in pediatric solid tumors whereas few evidences suggest his involvement in the pathogenesis of haematologic malignancies. Here we show that MYCN is overexpressed in a relevant proportion (40 to 50%) of adult and pediatric T-cell acute lymphoblastic leukemias (T-ALL). Focusing on pediatric T-ALL, MYCN-expressing samples were found almost exclusively in the TAL1-positive subgroup. Moreover, TAL1 knockdown in T-ALL cell lines resulted in a reduction of MYCN expression, and TAL1 directly binds to MYCN promoter region, suggesting that TAL1 pathway activation could sustain the up-regulation of MYCN. The role of MYCN in T-ALL was investigated by peptide nucleic acid (PNA-MYCN)-mediated transcriptional silencing of MYCN and by siRNAs. MYCN knockdown in T-ALL cell lines resulted in a reduction of cell viability, up to 50%, while no effect was elicited with a mismatch PNA. The inhibitory effect of PNA-MYCN on cell viability was due to a significant increase in apoptosis. PNA-MYCN treatment in pediatric T-ALL samples reduced cell viability of leukemic cells from patients with high MYCN expression, while no effect was obtained in MYCN-negative blast cells. These results showed that MYCN is frequently overexpressed in pediatric T-ALL and suggested his role as a candidate for molecularly-directed therapies.

## INTRODUCTION

T- cell acute lymphoblastic leukemia (T-ALL) represents about 15% of pediatric ALL cases and is generally associated with unfavorable clinical features and aggressive biologic behavior such as higher risk for primary resistant disease, early relapse and isolated central nervous system relapse compared with B-progenitor ALL patients. The prognosis of T-ALL in children and adolescent has improved in recent years as a result of more intensive chemotherapy approaches but it remains worse compared to B-lineage acute leukemia especially in presence of poor initial response to therapy [1-3].

Current understanding of the molecular pathogenesis of T-ALL has allowed to identify several oncogenes aberrantly expressed in different subgroups of pediatric T-ALL, such as TLX1, TAL1, LYL1, TLX3 and HOXA genes [4]. Even if many studies have reported promising results [5-9], unfortunately the improvement in understanding the biological background of pediatric T-ALL has not yet triggered a comparable development of novel molecularly-directed therapies that may improve patients' prognosis.

MYCN belongs to the MYC family of proto-oncogenes which encode for transcription factors involved in the control of cell proliferation, differentiation and apoptosis and broadly implicated in oncogenesis [10, 11]. During murine development MYCN expression is high in multiple tissues but it becomes down-regulated over the first weeks of life, persisting mainly in the early phases of B-cell differentiation [12, 13]. MYCN is overexpressed, frequently as a consequence of genomic amplification, in a large number of childhood malignancies, such as neuroblastoma, rhabdomyosarcoma and medulloblastoma, and enhanced MYCN expression correlates with increased growth potential and dismal prognosis [14-18]. Even if the mechanism through which MYCN mediates tumorigenesis remains largely unknown, experimentally induced downregulation of MYCN in neuroblastoma cells leads to growth arrest and cell death *in vitro* and decreased incidence and tumor mass *in vivo* [19, 20].

Up to now few evidences have suggested a possible involvement of MYCN in the pathogenesis of haematologic malignancies. For example, MYCN locus is a common target of retroviral integration in mouse T-cell lymphoma [21], and transgenic mice that overexpressed MYCN (Eμ-N-myc genes) develop pre-B and B lymphoid malignancies. The Eμ-N-myc transgene also appears to participate in the generation of T cell lymphoma [22]. High MYCN expression was detected in five of six acute myeloid leukemia (AML) cases, in one of nine acute lymphoblastic leukemia (ALL) cases and in several leukemia cell lines [23, 24]. MYCN overexpression was observed in a relevant percentage of pediatric patients with AML by micro-array and qRT-PCR analysis. Moreover, retrovirus-mediated overexpression of MYCN in mouse bone marrow stimulates the proliferation and self-renewal of myeloid cell *in vitro* and rapidly causes AML *in vivo* [25].

Peptide nucleic acids (PNA) are DNA analogs with considerable therapeutic potential [26]. Our group has already achieved interesting results by using PNA-mediated inhibition of MYCN in neuroblastoma cell lines [19, 27] while other studies supported the possible applications of anti-gene oligonucleotide strategies in hematologic malignancies [28, 29]. Recently we showed that a pharmacological inhibition of MYCN through administration of a specific PNA (PNA-MYCN) is able to block rhabdomyosarcoma cell growth both *in vitro* and *in vivo* , without evidence of systemic toxicity [30].

We show here that MYCN oncogene is overexpressed in a relevant proportion of pediatric T-ALL, in particular in the TAL1+ subgroup, and its selective inhibition by using PNA targeted against MYCN results in the arrest of cell growth and induction of apoptosis both *in vitro* and in human blast cells from pediatric T-ALL patients.

## RESULTS

### MYCN expression in pediatric T-ALL

**Figure 1:**
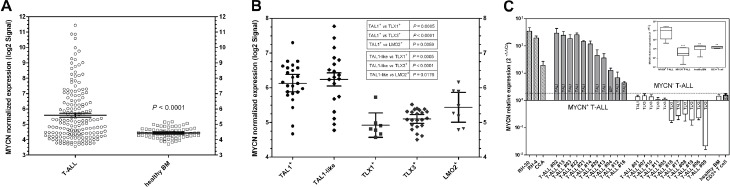
MYCN expression in T-ALL (A, B) MYCN normalized expression expressed in the log2 scale derived from the Stage 1 Microarray Innovations in LEukemia (MILE) study (GSE13159) 31 (A) and the pediatric *SET-NUP214* T-ALL dataset (GSE10609) 32 (B). For each group the expression level of each sample and the mean ± 95%CI are shown. (A) MYCN expression in 174 adult and pediatric T-ALL and 74 bone marrows from healthy donors. (B) data taken from the *SET-NUP214* dataset (80 pediatric T-ALL samples) that includes 24 TAL1+, 22 TLX3+, 7 TLX1+, 8 LMO2+ cases and 19 without any recurrent cytogenetic aberration but showing a TAL1-like gene signature (TAL1-like). (C) qRT-PCR analysis of MYCN expression in leukemic blast cell from 22 T-ALL pediatric patients and from healthy donor bone marrows and CD3+ T cells. DDCt method was used to quantify gene product level relative to three housekeeping genes: GAPDH, ATP5B and ACTB. T-ALL samples were considered MYCN-positive if the expression level was higher that the mean +3SD of that in healthy bone marrow donors and CD3+ T cells from healthy individuals and if showing statistical significance. In 11 out of 22 patients (50%) MYCN expression is significantly higher than healthy bone marrow donors. MYCN expression in two MYCN-amplifed cell lines (RH-30 and RH-4) and in one MYCN-overexpressing cell line (CCA) are shown for comparison. The embedded boxplot shows results of the statistical analysis of data distribution in the different subgroups. Student *t*-test: ***, *P*<0.0001; **, *P*<0.01.

To assess the frequency of MYCN overexpression in T-ALL we analyzed the expression profiles of blast cells from 174 pediatric and adult T-ALL samples and of mononuclear cells from 74 healthy bone marrow donors from the previously reported Stage 1 MILE study [31]. MYCN expression in T-ALL samples was significantly higher than that in bone marrow from healthy individuals (mean expression ± 95% CI: 5.57 ± 0.25, T-ALL; 4.44 ± 0.08, healthy donor BM; P< 0.0001, Student t test, two-tailed) and 40% of T-ALL samples expressed MYCN at a level higher than the mean + 3SD of expression in normal bone marrow (Fig. 1A).

Focusing on pediatric T-ALL, we analyzed the expression profiles of blast cells from the *SET-NUP214* dataset [32] finding that MYCN-expressing samples were found almost exclusively in the TAL1+ subgroup (Fig. 1B), while the other molecular subgroups did not show almost any MYCN overexpression (mean expression ± 95% CI: 6.13 ± 0.25, TAL1+; 4.92 ± 0.35, TLX1+; 5.09 ± 0.13, TLX3+; 5.43 ± 0.42, LMO2+). The same was true for the class of patients without recurrent cytogenetic aberrations that the authors reported to show a TAL1-like gene expression profile; these patients exhibited the same upregulation of MYCN expression as those carrying rearrangements of the TAL1 locus (Fig. 1B).

To further assess the expression of MYCN in pediatric T-ALL, we determined MYCN expression level in blast cells from 22 pediatric patients treated at the Pediatric Oncology and Hematology Section, Policlinico S.Orsola-Malpighi of Bologna, by qRT-PCR (Fig. 1C). In 11 out of 22 samples (50%) MYCN expression was significantly higher than that in bone marrow donors and CD3+ T-cells from healthy individuals (*P*= 0.001 and *P*= 0.005 respectively, Student t test, two-tailed). Single nucleotide polimorphism analysis (SNParray) and FISH analysis performed on the samples with higher MYCN expression revealed that MYCN overexpression was not due to amplification of the gene (Supplementary Figure 1).

### Clinical and biological features associated with MYCN expression

MYCN expression did not correlate significantly with overall and event-free survival while it was associated with poor response to steroid prephase (P=0.009, Fisher exact test). To confirm the association with different oncogene subclasses we analyzed TAL1, TLX1 and TLX3 positivity in leukemic cells from the 22 pediatric T-ALL patients by qRT-PCR. Positivity for TAL1, TLX1 and TLX3 was detected in 11 (50%), 2 (9%) , and 7 (32%) samples respectively (Supplementary Figure 2). Among the TAL1-positive patients we did not find any translocation leading to TAL1 activation, while six out of eleven patients showed evidence of the interstitial deletion leading to SIL-TAL1 fusion transcript (Supplementary Figure 3). In line with gene expression profiling results, MYCN overexpression was significantly found only in the TAL1+ group, since almost all the TAL1+ samples overexpressed MYCN, while no TLX1+ or TLX3+ samples showed high level of MYCN expression. Positive association between TAL1 and MYCN overexpression was statistically significant (*P*= 0.009, Fisher exact test), as well as inverse association between MYCN and TLX3 (*P*= 0.004).

The high MYCN expression in a considerable proportion of pediatric T-ALL suggested its potential role as a target for novel molecularly-directed therapies. Therefore we examined MYCN gene and protein expression level in human T-ALL cell lines (Jurkat, CCRF-CEM, RPMI 8402 and DND-41) to be used as in vitro models. All cell lines apart from DND-41 to be used as *in vitro* models. All cell lines apart from DND-41 showed high MYCN transcript and protein expression, at levels comparable to other cell lines known to overexpress MYCN (CCA rhabdomyosarcoma cell line) although lower than MYCN-amplified cell lines (RH30 rhabdomyosarcoma cell line) (Supplementary Figure 4A,B). It is noteworthy that the first three cell lines overexpress TAL1, with RPMI 8402 and CCRF-CEM also showing cryptic 1p32 deletion that gives rise to SIL-TAL1 fusion (Supplementary Figure 3B), while DND-41 harbours *t*(5;14)(q35.1;q32.2) that activates TLX3 .

**Figure 2:**
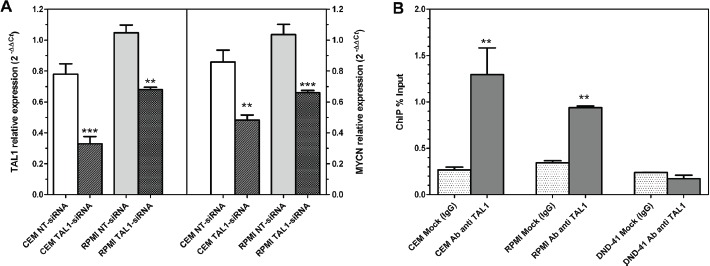
TAL1 effect on MYCN expression (A) qRT-PCR analysis of TAL1 (left) and MYCN (right) expression in CCRF-CEM and RPMI-8402 cells after 24 hours from nucleofection with siRNA targeted against TAL1 with respect to control non-targeting siRNA (NT-siRNA). No effect is elicited in cells treated with NT-siRNA. (B) ChIP analysis of MYCN promoter region: immunoprecipitation with a TAL1-specifc antibody significantly enriches the promoter region upstream of MYCN gene with respect to an irrelevant antibody (IgG) in TAL1-positive cells (CCRF-CEM and RPMI-8402) and not in TAL1-negative (DND-41). Data were analyzed by the percent input method. Student t-test: ***, *P*<0.0001; **, *P*<0.01.

To further investigate the possible link between TAL1 and MYCN concurrent overexpression observed in T-ALL cell lines and leukemic blast cells, CCRF-CEM and RPMI-8402 cells were transfected with siRNAs targeted against TAL1. After 24 hours from nucleofection, TAL1 knockdown by siRNAs resulted in a significant reduction of MYCN expression, implying that TAL1 pathway activation could directly or indirectly lead to a significant increase in MYCN expression (Fig. 2A). Moreover, ChIP analysis showed that MYCN can be directly targeted by TAL1, since the promoter region upstream of MYCN is significantly enriched by immunoprecipitation with a TAL1-specifc antibody in TAL1-positive cell lines (CCRF-CEM and RPMI-8402) but not in the TAL1-negative DND-41 cell line (Fig. 2B).

### Effect of MYCN knockdown in T-ALL blast cells and cell lines

**Figure 3:**
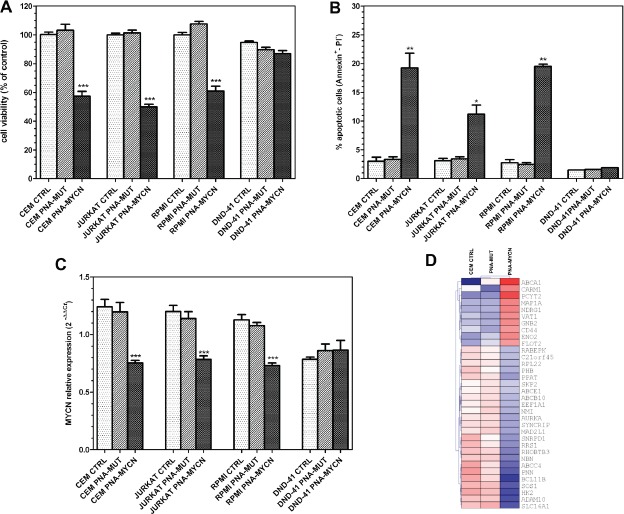
Effect of PNA-mediated MYCN silencing in T-ALL cell lines PNA-MYCN effect was analyzed in MYCN-positive cell lines (CCRF-CEM, Jurkat and RPMI-8402); MYCN-negative cell line DND-41 is shown for comparison. (A) Cell viability assessed after 72 hours from seeding with ATPLite assay. MYCN knockdown in CCRF-CEM, JURKAT, RPMI 8402 by PNA-MYCN treatment results in a reduction in cell viability of 42%, 50% and 39% respectively. No reduction in viability is observed in cells treated with mismatch PNA (PNA-MUT). (B) Flow-cytometric analysis of apoptotic cells measured by Annexin-V/PI staining performed in CCRF-CEM, JURKAT and RPMI 8402 cell lines. PNA-MYCN treatment leads to increased apoptosis with respect to control and PNA-MUT treatment. (C) qRT-PCR analysis on CCRF-CEM, JURKAT and RPMI 8402 show significant reduction in MYCN gene expression, whereas no effect is obtained with PNA-MUT. To allow the simultaneous representation of gene expression differences in all cell lines, including DND-41 that do not express MYCN, data were normalized to the mean expression level of the cell line. Student t-test: ***, *P*<0.0001; **, *P*<0.01; *, *P*<0.05. (D) Heatmap representation of MYCN target genes as derived from the MYCNot database that are induced or repressed by MYCN inhibition in CCRF-CEM cell line by PNA-MYCN treatment.

To investigate if MYCN could be an oncogenic target in T-ALL, we inhibited MYCN expression in T-ALL cell lines by a peptide nucleic acid (PNA) targeted against MYCN transcription (PNA-MYCN) [19, 27, 30]. After 72 hours of treatment, MYCN knockdown by PNA-MYCN on CCRF-CEM, Jurkat and RPMI 8402 resulted in a significant reduction of cell viability of 42%, 50% and 39%, respectively (*P*<0.0001, Student t test, two-tailed) (Figure 3A). No effect was elicited by using a mismatch PNA (PNA-MUT) harboring 3 point mutations, thus demonstrating the mechanism specificity. Furthermore PNA-MYCN treatment was not effective in the MYCN-negative DND-41 cell line (Fig. 3A). Flow cytometric analysis of Annexin-V/PI staining performed on the three cell lines showed that the inhibitory effect of anti-gene PNA-MYCN on cell viability led to increased apoptosis of 19.3%, 11.2% and 19.5% in CCRF-CEM, Jurkat and RPMI 8402, respectively, while no effect was elicited on the MYCN-negative cell line (Fig. 3B). PNA-MYCN treatment led to significant down-regulation of MYCN in the three cell lines, with no effect of control PNA-MUT (Fig. 3C). MYCN down-regulation in T-ALL cell lines was not associated with a reduction of TAL1 gene expression, suggesting the absence of a positive loop between TAL1 and MYCN (data not shown). PNA-MYCN effect on cell viability, on MYCN expression and on apoptosis was specific, since no effect was obtained with PNA-MUT treatment. MYCN inhibition in CCRF-CEM cell line determined the differential expression of known MYCN target genes derived from the MYCNot database, in particular upregulation of N-MYC downstream regulated gene 1 (NDRG1) and CD44, that are normally repressed by MYCN, and downregulation of SLC16A1, hexokinase 2, ABCC4 and ABCE1, ADAM10 and BCL11B (Fig. 3D). Overall these genes regulate leukocyte activation (NBN, ADAM10, RPL22, BCL11B, NDRG1), progression through cell cycle (MAD2L1, SKP2, RRS1, C21orf45, AURKA), glycolysis (ENO2, HK2) and drug transport (ABCC4, ABCB10, ABCE1).

To further demonstrate the specificity of the biological effect mediated by MYCN, we inhibited MYCN expression also by RNAi on RPMI-8402 cell line, confirming that MYCN downregulation significantly inhibits cell growth (Supplementary Figure 5A). The effect however is smaller than the one elicited by PNA-MYCN (24% and 37% inhibition at 24 and 48 hours), since RNAi are less efficient that PNAs at inhibiting MYCN (Supplementary Figure 5B).

**Figure 4:**
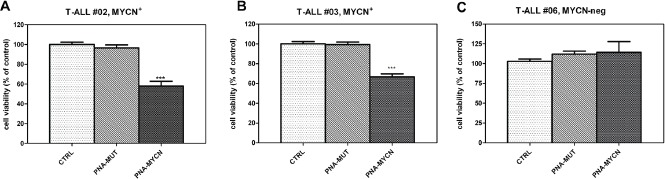
Effect of PNA-MYCN and PNA-MUT treatment on T-ALL leukemic blast cells from pediatric patients Cell viability is reduced in two patients with high MYCN expression (A,B) whereas no inhibition is found in one MYCN-negative patient (C). No effect is elicited with PNA-MUT. Student t-test: ***, *P*<0.0001 both vs. control and PNA-MUT.

To assess the efficacy of MYCN inhibition as a potential therapeutic strategy in T-ALL, we examined pediatric T-ALL patient samples isolated from bone marrow or peripheral blood for their sensitivity to PNA-MYCN. PNA-MYCN treatment significantly reduced cell viability of blast cells from two pediatric patients with high MYCN expression (42% for T-ALL#02 and 34% for T-ALL#03) (*P*<0.0001), while no effect was elicited on leukemic cells from one MYCN-negative patient (T-ALL#06) and by using the mismatch PNA (PNA-MUT) (Fig. 4).

## DISCUSSION

The implication of MYCN in the genesis of T-cell leukemias and lymphomas was suggested by few previous studies on the evidence of high MYCN expression in neoplasms arisen in transgenic mice or found in leukemia and lymphoma cell lines [21-25]. In this article we show that MYCN could play a role in the oncogenic phenotype of T-lineage ALL: as a matter of fact we find that 40 to 50% of T-ALL overexpress MYCN, in close association with the overexpression of TAL1. These findings highlight that another member of the myc-family of trascription factors could be a critical component of T-ALL oncogenesis, along with MYC which has already been characterized as a master oncogene in human T-ALL, particularly as a consequence of Notch1 mutation [33-35]. However, the mechanism through which MYCN mediates oncogenesis in T-ALL has yet to be precisely defined. On the basis of the concurrent overexpression of MYCN and TAL1 detected in a large proportion of blast cells from pediatric T-ALL samples, along with the evidence in qRT-PCR analysis that TAL1 knockdown resulted in a reduction on MYCN expression and that TAL1 directly binds to MYCN promoter region by ChIP analysis, we hypothesized that TAL1 pathway activation could directly sustain the up-regulation of MYCN expression. The work by Kusy *et al.* [36] lends further support to this hypothesis evaluating the gene expression profile of two T-ALL cell lines in basal conditions and after TAL1 or NKX3.1 silencing by RNA interference: in the cell line showing high levels of TAL1 protein (Jurkat), knockdown of TAL1 or of its downstream effector NKX3.1 significantly down-regulates MYCN expression (derived from the analysis of published gene expression data). On the other hand, the fact that MYCN down-regulation is not associated with a reduction in TAL1 expression suggests the absence of a positive loop between MYCN and TAL1.

**Table 1:** Clinical data of the patients included in the study. CCR: Continued Complete Remission; ETP: early T cell precursor; GPR: Good Prednisone Responder; PPR: Poor Prednisone Responder; HR: High risk; MR: Medium risk; NA: not available; REL: relapsed; SR: Standard risk; †: death;. Patients enrolled in AIEOP protocols ALL 95 e CCG 1901 do not show MRD evaluation, that was included in ALL2000 protocol

Patient ID	AIEOP protocol	Age (yrs)	Sex	WBC/mm3 at diagnosis	Immuno-phenotype	Prednisone response	MRD	Follow-up (months of observation)
T-ALL #01	ALL 2000	6	M	130.000	Intermediate	GPR	MR	CCR(120)
T-ALL #02	ALL 2000	16	M	153.000	Intermediate	PPR	HR	CCR (84)
T-ALL #03	ALL 2000	12	M	180.000	Early-T	PPR	HR	REL-†(16)
T-ALL #04	ALL 2000	6	F	43.000	ETP	PPR	MR	REL-†(18)
T-ALL #05	ALL 2000	5	M	155.000	Early-T	GPR	HR	CCR (82)
T-ALL #06	ALL 2000	5	M	310.000	Intermediate	GPR	HR	REL-†(35)
T-ALL #07	ALL 2000	6	M	42.000	Early-T	PPR	MR	CCR (61)
T-ALL #08	ALL 2000	7	M	200.000	Intermediate	GPR	SR	CCR (60)
T-ALL #09	ALL 2000	10	M	87.000	Mature	GPR	MR	CCR (96)
T-ALL #10	ALL 2000	6	M	556.000	Intermediate	PPR	MR	REL-†(28)
T-ALL #11	ALL 2000	5	M	560.000	Early-T	GPR	MR	REL-†(100)
T-ALL #12	ALL 2000	10	M	1250	Early-T	GPR	MR	CCR(99)
T-ALL #13	ALL 2000	4	M	78.000	Early-T	GPR	HR	CCR (120)
T-ALL #14	ALL 2000	11	M	400.000	Intermediate	PPR	HR	REL-†(9)
T-ALL #15	ALL 2000	8	F	45.000	Mature	PPR	MR	CCR (58)
T-ALL #16	ALL 95	3	F	90.000	Mature	PPR	NA	CCR (179)
T-ALL #17	ALL 95	2	M	510.000	Mature	GPR	NA	CCR(188)
T-ALL #18	ALL 95	9	M	80.000	Mature	GPR	NA	CCR (177)
T-ALL #19	ALL 95	9	M	290.000	Mature	GPR	NA	REL (140)
T-ALL #20	CCG 1901	9	M	40.000	NA	NA	NA	REL-†(18)
T-ALL#21	ALL 2009	12	F	160.500	Early-T	PPR	MR	CCR (15)
T-ALL#22	ALL 2009	10	F	138.200	Early-T	PPR	NA	CCR (1)

Previously Ferrando *et al.* reported that MYCN can be overexpressed in T-ALL, in particular in the LYL1+ subgroup [4]. It is interesting to note that both TAL1 and LYL1 belong to the basic helix-loop-helix (bHLH) family of transcription factors which are implicated in T-cell leukemogenesis mainly by interfering with the transcriptional activity of E2A and HEB during T-cell lymphopoiesis [37-40]. This fact suggests that they could act through a common mechanism in increasing MYCN expression and supports the idea that different pathways can converge on MYCN activation. In fact, we observed that not only patients carrying SIL-TAL1 fusion show MYCN overexpression, but also those with TAL1 overexpression without any TAL1 rearrangement, or those exhibiting a TAL1-like signature (data from the Dutch SET-NUP214 dataset). We know from the literature that up to 60% of T-ALL patients display TAL1 abnormal expression, and that other mechanisms, apart from chromosomal rearrangement or interstitial deletion, lead to TAL1 upregulation [41]. This would imply that MYCN could be an interesting therapeutic target in a large proportion of patients with T-ALL and it is plausible that other oncogenic events or phenotypic T-ALL classes (as the early T cell precursor ALL - ETP-ALL) can exhibit MYCN activation. Indeed we show here that a case of ETP-ALL (T-ALL#04) overexpresses MYCN, further sustaining the hypothesis that MYCN upregulation can be a common event in T-ALL without TLX1 or TLX3 abnormalities. Whether gaining further insights on the molecular pathways that could link MYCN and TAL1 expression is needed, these results indicates that MYCN is a potential component of pediatric T-ALL genesis and a candidate for targeted therapy. Considering this assumption, in this study we investigated the ability of nucleic acid analogs targeted against MYCN transcription to impair viability of blast cells from two pediatric patients with T-ALL overexpressing MYCN. We found that selective MYCN inhibition resulted in a significant reduction of blast cells viability *ex vivo.* An encouraging impairment of cell growth and viability, mainly through the activation of the apoptotic pathway, was also observed in T-ALL cell lines. These findings indicate that PNA-mediated transcriptional silencing of MYCN could significantly inhibit T-ALL cell growth both *in vitro* and on primary leukemic cells, which supports the role of MYCN as a target for therapy in T-ALL. Moreover, our results highlight peptide nucleic acids as anti-gene agents with considerable therapeutic potential complementary to conventional therapies not only in pediatric solid tumors [30] but also in hematopoietic malignancies. More preclinical studies evaluating the effect of MYCN inactivation will be necessary to determine whether this oncogene can be effectively exploited as a novel target for therapy of pediatric T-ALL.

## METHODS

### Patient samples

Bone marrow or peripheral blood-derived leukemic cells were collected (with informed consent in accordance with the Declaration of Helsinki and institutional review board approval) from children with T-ALL who were treated with Berlin-Frankfurt-Muenster (BFM) -oriented chemotherapy being enrolled in AIEOP ALL 95, 2000 or 2009 protocols at the Oncology and Hematology Section of the Pediatrics Operative Unit, Policlinico S. Orsola-Malpighi, Bologna. Mononuclear cells were purified by Ficoll-Hypaque centrifugation before cryopreservation. All specimens consisted of more than 85% lymphoblasts. Clinical features of the patients are summarized in Table 1. Immunophenotypic, cytogenetic and molecular analysis was performed according to the relevant Protocol.

### Gene expression analyses

Gene expression profiles of primary T-ALL patient samples and healthy normal bone marrow samples were derived from two previously published datasets stored at the NCBI Gene Expression Omnibus database: the Stage 1 Microarray Innovations in LEukemia (MILE) study [31] (GSE13159) and the pediatric *SET-NUP214* T-ALL dataset that includes TLX3-, TLX1-, TAL1-, LMO2-and HOXA-positive molecular subgroups (GSE10609) [32]. Gene expression profiles from a total of 174 adult and pediatric T-ALL and 74 bone marrows from healthy donors were taken from the first study; from the second study we selected 61 pediatric T-ALL samples including 24 TAL1+, 22 TLX3+, 7 TLX1+ and 8 LMO2+ cases and 19 samples of unknown class showing a TAL1-like gene expression signature. Samples belonging to classes of ≤ 5 patients were excluded from the analysis. Raw data (CEL files) of hybridization to HG-U133 Plus 2.0 arrays (Affymetrix) were normalized and summarized with the *rma* algorithm using Bioconductor packages under the R environment.

### Cell lines and apoptosis assay

T-ALL cell lines were cultured in RPMI-1640 medium (Invitrogen, Milan, Italy) supplemented with 10% fetal calf serum (Euroclone, Milan, Italy), 100 IU/mL penicillin, 100 μg/mL streptomycin and 1% L-glutamine, at 37°C in humidifed air containing 5% CO_2_.

To assess the extent of apoptosis induction, flow cytometric analysis of Annexin V–FITC/propidium iodide (PI)-stained samples was performed after 6 hours of treatment with PNA-MYCN, PNA-MUT and in basal condition, following manufacturer's instruction (Annexin-V-FLUOS Staining Kit, Roche Diagnostics, Monza, Italy). Samples were analyzed by a FACSCanto flow cytometer (Beckton Dickinson, Franklin Lakes, NJ). Apoptotic cells were identified as Annexin V-positive, PI-negative cells.

### PNA and siRNA treatment

The anti-gene PNA either specific for the MYCN gene (PNA-MYCN) or containing three point mutations in the PNA-MYCN sequence to check specificity (PNA-MUT), were designed as previously reported and conjugated to a nuclear localization signal (NLS) peptide for the delivery [19, 30]. The PNA-peptides were synthesized using solid phase peptide synthesis protocols as previously described [19, 30], and purified by preparative HPLC (using IP-RP methodology); the purified products were analyzed by HPLC-ESI/MS to check purity.

For PNA experiments, cells were seeded in 96-well plates at a concentration of 10^5^ cells/ml in Opti-MEM medium (Invitrogen) supplemented with antibiotics and glutamine, and treated with PNA-MYCN or PNA-MUT at a final concentration of 10μM. Fetal calf serum (4%) was added after 6 hours of incubation with PNA. Cell viability was measured 72 hours after seeding with ATPlite assay (PerkinElmer, MA, USA). For each cell line, different treatments were performed in triplicate and each experiment was replicated at least 4 times.

For the analysis of primary patient samples, cryopreserved T-ALL lymphoblasts were thawed and viable cells were counted with acridine orange. 5 × 10^5^ viable cells were plated in Opti-MEM medium supplemented with 100 IU/mL penicillin, 100 μg/mL streptomycin, 1% L-glutamine, gentamycin, amphotericin-B, Insulin/Transferrin/Sodium Selenite (Sigma Aldrich, Milan, Italy) and treated with PNA-MYCN or PNA-MUT at a final concentration of 10μM. Fetal calf serum was added after 6 hours, at a final concentration of 20%. Cell viability was measured after 96 hours of incubation, by acridine orange counting and ATPlite assay. At least five wells for each treatment were seeded.

TAL1 inhibition was performed by nucleofection on a Nucleofector II device (Lonza GmbH, Cologne, Germany) using Hs_TAL1_5 and Hs_TAL1_6 siRNAs (Qiagen, Milan, Italy). MYCN inhibition was performed with the small interfering RNA MYCN_7 (5'-GGACAGUGAGCGUCGCAGAUU-3') purchased from Dharmacon Research (ThermoScientific, Lafayette, Colorado). Mock transfection was performed with a non-targeting (NT) control siRNA (Dharmacon Research). CCRF-CEM and RPMI-8402 cells were transfected with siRNAs at a final concentration of 50-200 nM and the effect on TAL1 and MYCN expression was analyzed 24-48 hours after nucleofection.

### RNA extraction and quantitative PCR (qRT-PCR)

Total RNA was extracted by either RNeasy spin column method (Qiagen) or TriZol reagent (Invitrogen) following manufacturer's instructions after 6 hours of treatment with PNA-MYCN, PNA-MUT and in basal condition. 1 μg total RNA was reverse transcribed to single-stranded cDNA using the Transcriptor first strand cDNA synthesis kit (Roche Diagnostics) with oligo-dT primers (2.5 μM). Gene-specific primers amplifying MYCN, TAL1, TLX1 and TLX3 were designed with Primer Express 3.0 Software (Applied Biosystems, Monza, Italy) and qRT-PCR was performed using FastStart Sybr Green (Roche Diagnostics) on the LightCycler 480 apparatus (Roche Diagnostics). DDCt method was used to quantify gene product levels relative to three housekeeping genes, GAPDH, ATP5B and ACTB. Quantitative RT-PCR primer sequences were as follows: MYCN-forward, 5'-CGACCACAAGGCCCTCAGT-3'; MYCN-reverse, 5'-TGACCACGTCGATTTCTTCCT-3'; TAL1-forward, 5'-TTCACCACCAACAATCGAGTG-3'; TAL1-reverse, 5'-CCGCGTTCACATTCTGCTG-3'; TLX1-forward, 5'-CCCTGGATGGAGAGTAACCG-3'; TLX1-reverse, 5'-CAGGCGTGTGAAGGACGTG-3'; TLX3-forward, 5'-TTCCAAAACCGGAGGACCA-3'; TLX3-reverse, 5'-ACAGCGGGAACCTTGGAAC-3'; GAPDH forward, 5'-CGGGAAGCTTGTCATCAAT-3'; GAPDH reverse, 5'- GACTCCACGACGTACTCAGC-3'; ATP5B forward, 5'-GTCTTCACAGGTCATATGGGGA-3'; ATP5B reverse, 5'-ATGGGTCCCACCATATAGAAGG-3'; ACTB forward, 5'-TCCTCCCTGGAGAAGAGCTACGAG-3'; ACTB reverse, ACACGGAGTACTTGCGCTCAGG-3'. All qRT-PCR primer pairs used span intron/exon boundaries. MYCN expression in T-ALL samples was compared to that in normal bone marrow and CD3+ T-cells from healthy individuals. Criteria for defining overexpression were: statistical significance by Student's *t* test and expression higher than the mean + 3SD of normal bone marrow and CD3+ T-cells.

SIL-TAL1 chimeric transcript, that results from the cryptic 1p32 deletion, was measured as previously described in the BIOMED-1 concerted action [42]. T-ALL samples were defined TAL1+ if expressing TAL1 at levels higher than the lowest expression found in T-ALL samples harboring TAL1 deletion, as previously described [4].

### SNP-array and FISH analysis

FISH experiments were performed on T-ALL cell lines with the Vysis specific probe protocols (Vysis, Downers Groove, USA) using the LSI-MYCN (2p24.1) probe marked with Spectrum Green (green), that contains unique DNA sequences specific for the MYCN oncogene located within the 2p24.1 region of chromosome 2, and the CEP2 probe, marked with Spectrum orange (red), complementary to sequences located in the centromeric region of chromosome 2 (2p11.1-2q11.1). FISH analysis to detect TAL1 rearrangements was performed on metaphase e interphase cells using two BlueFISH probes (BlueGnome Ltd., Cambridge) for chromosome 1 according to manufacturer's instructions.

Genomic DNA was extracted using the QIAamp DNA kit (Qiagen), labeled and hybridized to an oligonucleotide SNP array (Genome Wide SNP 6.0; Affymetrix, Santa Clara, CA, USA). Copy number analysis was performed by Partek Genomics Suite, which compares Signal log2 ratios to a reference of 270 normal Hapmap samples. Genomic segmentation was used to detect amplified and deleted segments with stringent parameters (*P* <0.0001, >15 markers, signal/noise ≤ 0.4). Multiple hypothesis correction by Benjamini and Hochberg was applied and FDR threshold was set at 0.05. SNP, gene and cytogenetic band locations are based on the *hg19* Genome build.

### Western blotting

Cells were collected by centrifugation, washed twice in phosphate buffered saline (PBS) and lysed for 30 min at 4°C in RIPA-buffer supplemented with 1 mM PMSF, 10μg/ml Aprotinin, 10μg/ml Leupeptin, 1 mM Orthovanadate Sodium salt (Sigma Aldrich). Homogenates were centrifuged at 13,000 × *g* for 15 min at 4°C and supernatants were stored at –80°C. Protein concentrations were determined with the BCA protein Assay (Pierce Rockford, IL). Ten micrograms of protein was resolved on a 10% SDS PAGE gel and transferred onto polyvinylidene difluoride (PVDF) membranes. Nonspecific binding sites were blocked by incubation in blocking buffer (PBS containing 0.1% Tween-20 (PBS/T), with 5% w/v BSA) for 1 hour at room temperature. After three PBS/T washes, membranes were incubated with primary antibodies overnight at 4°C in PBS/T with 5% bovine serum albumin. Then, membranes were washed and incubated with peroxidase conjugate secondary antibodies diluted in blocking buffer for 1 hour at room temperature. Antigens were revealed using Enhanced Chemiluminescence Reaction (ECL+, Amersham Pharmacia Biotech, Les Ulis, France). The following antibodies were used: mouse monoclonal MYCN antibody (Calbiochem, San Diego, CA, USA) and rabbit polyclonal β-Tubulin antiboby (Santa Cruz Biotechnology, Santa Cruz, CA).

### Chromatin Immunoprecipitation (ChIP)

Chromatin extracted from CCRF-CEM, RPMI-8402 and DND-41 cell lines was sonicated into fragments approximately 500bp in size. Immunoprecipitation was performed with Imprint Chromatin Immunoprecipitation Kit (Sigma-Aldrich, St Louis, MO, USA), using specific primary antibody against TAL1 (Santa Cruz Biotechnology, Inc.). RNA Polimerase II antibody or normal IgG were used as positive and negative controls.

The precipitated DNA fractions were then amplified by a Sybr-Green-based semi-quantitative Real-Time PCR method, using specific primers for putative MYCN promoter sequence (forward: 5΄-AGGCCTCCTCCCAGCACACTT -3΄; reverse: 5΄-GCTGCGGCCCAGAAATCTTCAGT -3΄). Reactions were run on the ABI PRISM 7300 sequence detection system (Applied Biosystems, Foster City, CA, USA). Data were normalized using the % of input (normalization to the amount of input chromatin) method [43].

### Gene expression profiling

CCRF-CEM cell line was treated with either PNA-MYCN or PNA-MUT at 10 µM for 12hr. Total RNA was then labelled following the Affymetrix GeneChip Expression Analysis Technical Manual (Affymetrix, Santa Clara, CA, USA) and hybridized onto HG-U133 Plus 2.0 Arrays (Affymetrix). Gene expression data were quantified by the MAS5 algorithm, filtered and differentially expressed genes were selected if called “Increased” or “Decreased” with respect to both untreated cells and cells treated with PNA-MUT by the Change algorithm of the MAS5 pipeline. MYCN target genes were selected from the MYCNot database [http://medgen.ugent.be/MYCNot], that lists all MYCN target genes. Hierarchical clustering using Manhattan distance was performed using MeV TM4 software [http://www.tm4.org].

### Statistics

The *t* -test was used to compare continuous variables obtained with *in vitro* and gene expression experiments. Differences in the distribution of categorical parameters were compared using Fisher's exact tests. A *p* -value of 0.05 was used as the cut-off below which results were considered statistically significant.

## Supplementary Figures and Tables




