# Supplementary material for: MYCN is a novel oncogenic target in pediatric T-cell Acute Lymphoblastic Leukemia
Source: Oncotarget. 2013 Sep 29;5(1):120–30. doi: 10.18632/oncotarget.1337 (PMC3960194; doi:10.18632/oncotarget.1337)
Supplement: Supplementary file 1 [file oncotarget-05-0120-s001.pdf]

Supplementary data

MYCN is a novel oncogenic target in pediatric T-cell Acute Lymphoblastic Leukemia

Annalisa Astolfi[1,2], Francesca Vendemini[2], Milena Urbini[1,2], Fraia Melchionda[2], Riccardo Masetti[2], Monica Franzoni[2], Virginia Libri[2], Salvatore Serravalle[2], Marco Togni[2], Giuseppina Paone[2], Daniela Bressanin[3], Francesca Chiarini[4], Alberto M. Martelli[3], Roberto Tonelli[5], Andrea Pession[1,2]

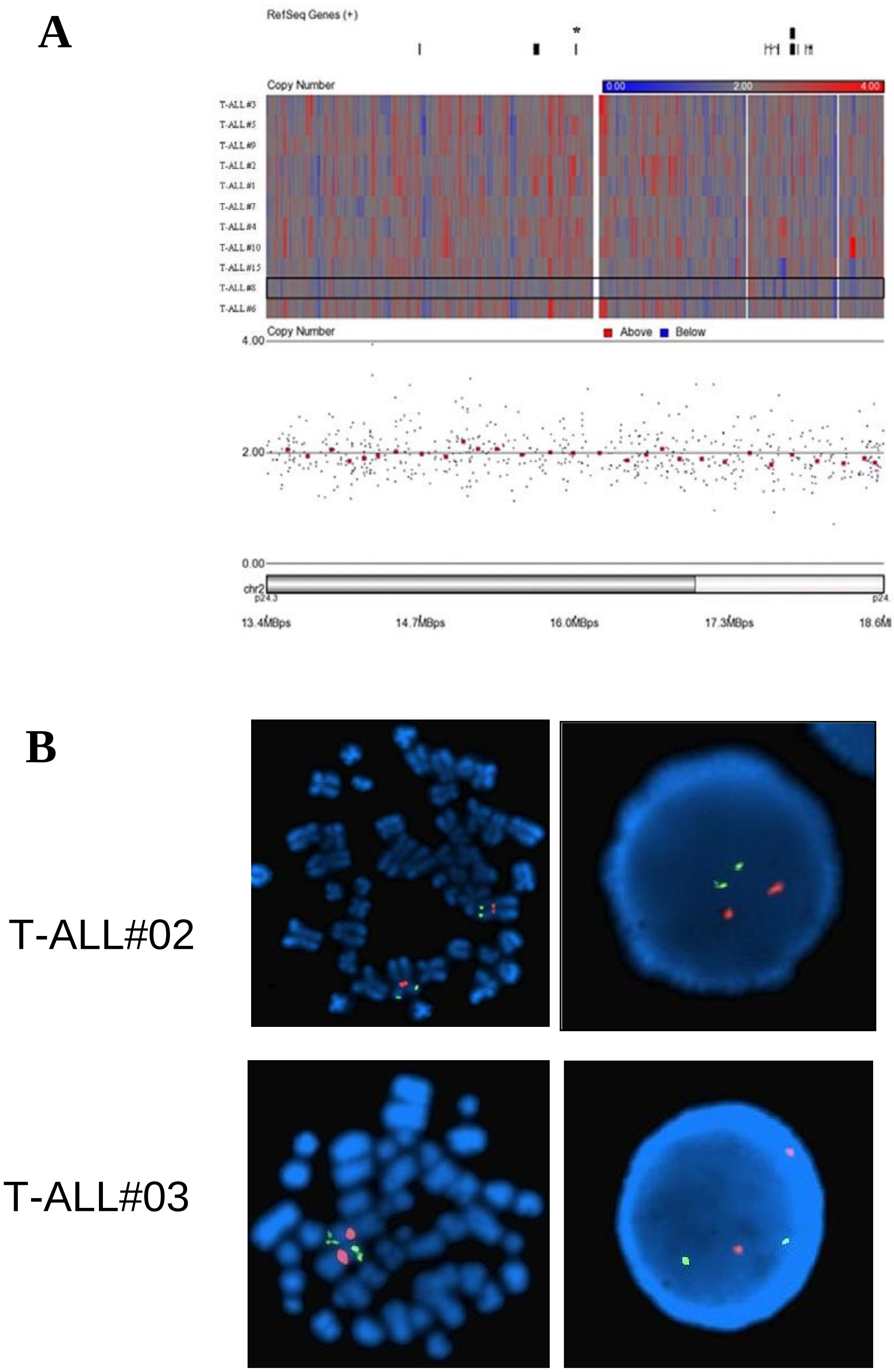

Supplementary Figure 1: (A) SNP-array analysis at the MYCN locus (identified by the asterisk). Above: Heatmap representation of the SNP copy number status in the MYCN region (*). Middle: copy number signal of one representative sample (T-ALL #08); red dots indicate copy number status smoothed on 30 adjacent markers. Below: cytoband representation where MYCN is located. **(B)** Fluorescence In-Situ Hybridization. Leukemic blast cells during metaphase and interphase from patients that overexpress MYCN. LSI-MYCN probe that recognizes the MYCN oncogene located within the 2p24.1 region is marked with Spectrum Green (green), while the CEP2 probe, complementary to sequences located in the centromeric region of chromosome 2, is marked with Spectrum orange (red). The presence of 4 signals in metaphase and 2 signals in interphase indicates that MYCN is present in diploid state.

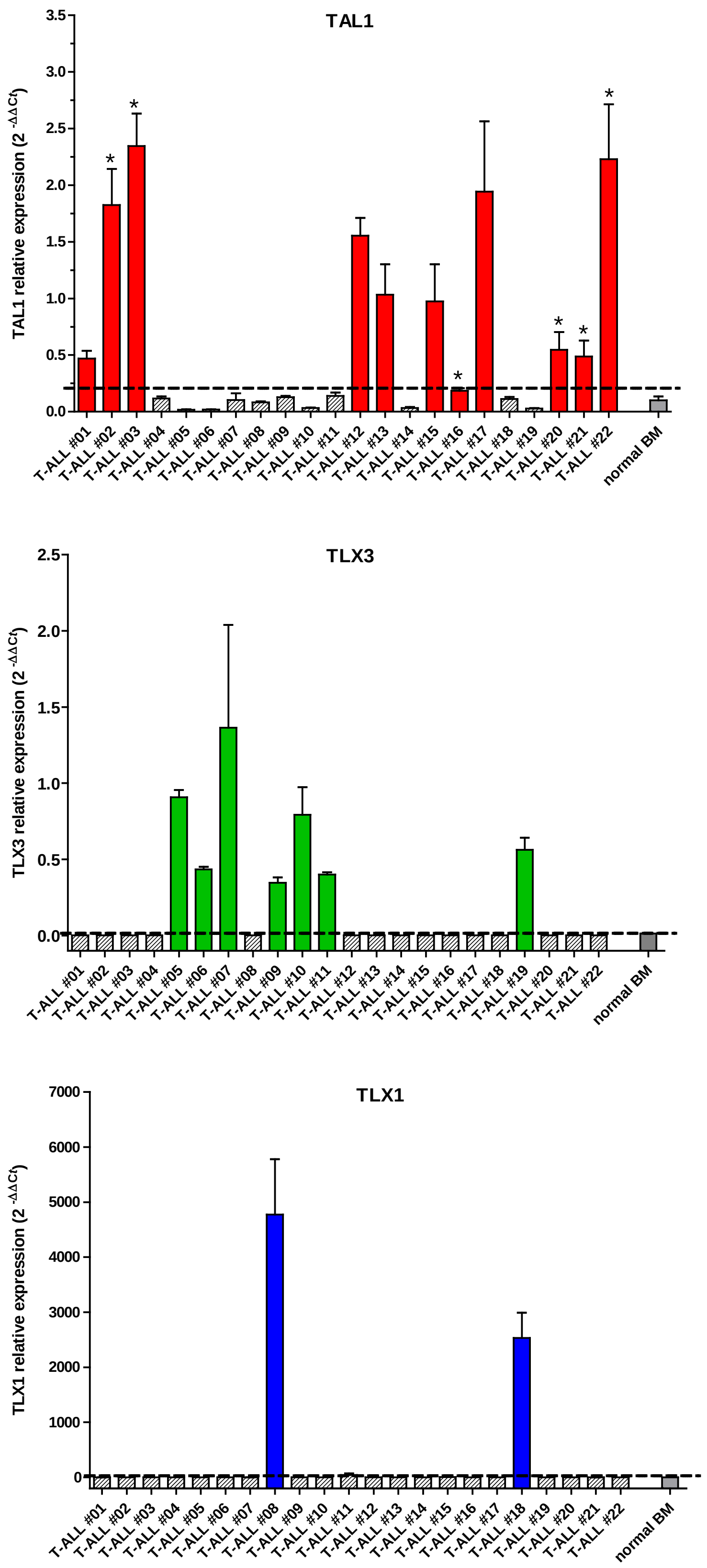

Supplementary Figure 2: Oncogene expression in leukemic blast cells, measured by qRT-PCR. Asterisks indicate samples with the SIL-TAL chimeric transcript (TALd1-positive samples), resulting from deletion of a 90 kb genomic DNA fragment adjacent to the TAL1 locus at the 1p32. Samples showing TAL1 expression levels above that detected in TALd1+ samples were considered TAL1-positive (red) **[4]**.

A

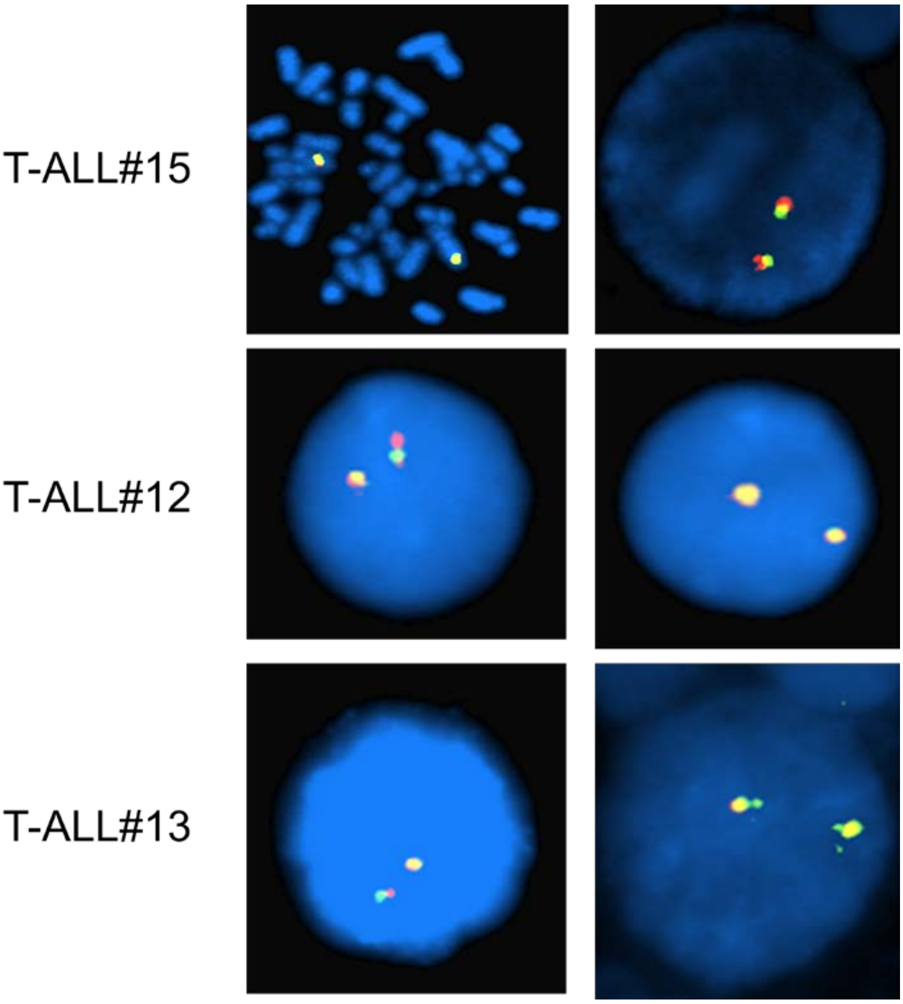

B

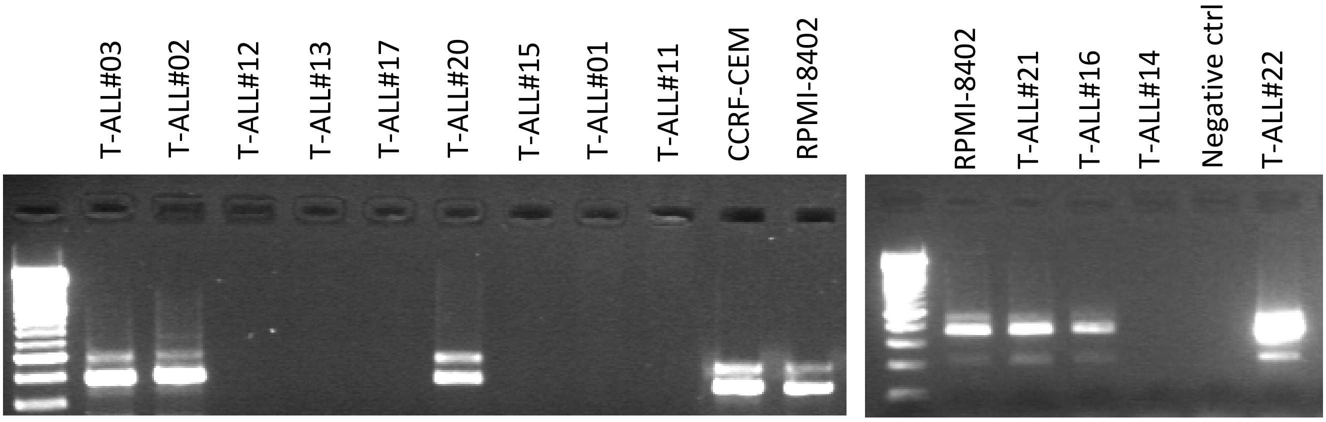

Supplementary Figure 3: TAL1 activation in T-ALL patient samples. (**A)** FISH analysis was performed using the two BAC clones RP11-346M5 (in red) and RP11-332M15 (in green), respectively upstream and downstream of TAL1 gene. All patients' metaphase and interphase nuclei show two orange/green fusion signal patterns indicating the absence of a translocation involving TAL1 gene. (**B)** PCR amplification of the SIL-TAL1 chimeric transcript that results from the 1p32 interstitial deletion.

A

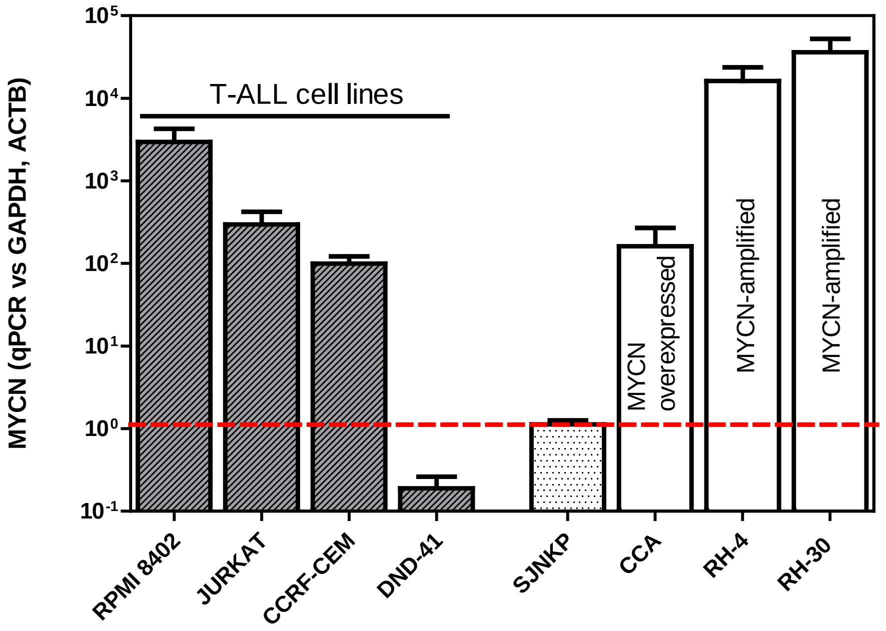

B

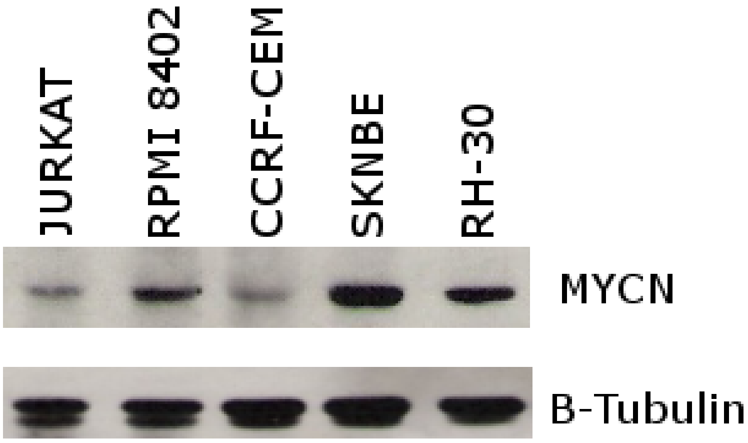

Supplementary Figure 4: MYCN expression in T-ALL cell lines. (**A**) qRT-PCR evaluation of MYCN expression in RPMI-8402, Jurkat, CCRF-CEM and DND-41 cell lines, compared with one negative control (SJNKP), and three positive controls (CCA, overexpressed; RH-4 and RH-30, amplified). (**B**) Western Blot assay. MYCN protein expression in Jurkat, RPMI-8402 and CCRF-CEM cell lines compared with two positive controls (RH-30 and SKNBE, amplified).

A

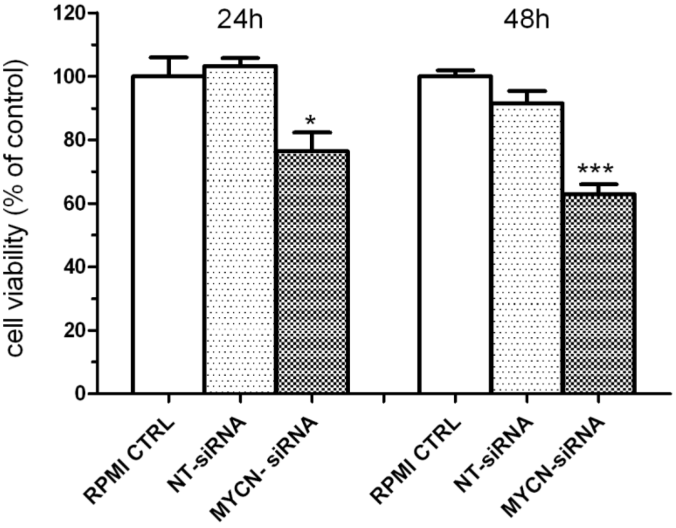

B

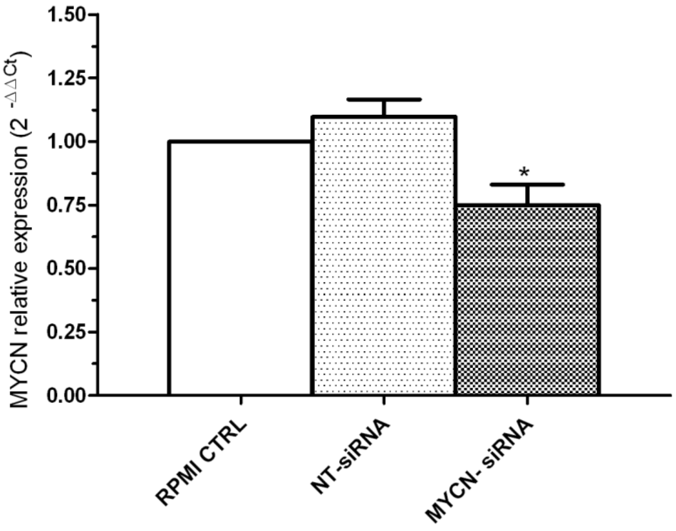

Supplementary Figure 5: **Effect of MYCN inhibition by RNA interference. (A)** Cell viability assessed after 24 or 48 hours from seeding with ATPLite assay. MYCN knockdown in RPMI 8402 by a specific siRNA results in a reduction in cell viability of 24% and 37% respectively. No reduction in viability is observed in cells treated with non targeting siRNA (NT). **(B)** qRT-PCR analysis shows significant reduction in MYCN gene expression after treatment with a specific siRNA directed against MYCN, whereas no effect is obtained with NT-siRNA. Student *t*-test: ***, P<0.0001; *, P<0.05.
